# Comparison of six assessment tools to screen for obstructive sleep apnea in patients with hypertension

**DOI:** 10.1002/clc.23714

**Published:** 2021-09-14

**Authors:** Zhenzhen Zheng, Xishi Sun, Riken Chen, Wei Lei, Min Peng, Xiongbin Li, Nuofu Zhang, Junfen Cheng

**Affiliations:** ^1^ Department of Respiratory and Critical Medicine The Second Affiliated Hospital of Guangdong Medical University Zhanjiang China; ^2^ Department of Respiratory and Critical Medicine The Affiliated Hospital of Guangdong Medical University Zhanjiang China; ^3^ Guangzhou Medical University, State Key Laboratory of Respiratory Disease, National Clinical Research Center for Respiratory Disease, Guangzhou Institute of Respiratory Health the First Affiliated Hospital of Guangzhou Medical University Guangzhou China

**Keywords:** Berlin questionnaire, ESS sleepiness scale, hypertension, no‐apnea score, NoSAS score, obstructive sleep apnea, STOP questionnaire, STOP‐bang questionnaire

## Abstract

**Background:**

Obstructive sleep apnea (OSA) is often accompanied by other complications, especially hypertension.

**Hypothesis:**

The purpose of this study is to compare the application value of six tools in the screening of OSA in patients with hypertension. Compared with other questionnaires, we hypothesized that Berlin performed better in screening hypertensive patients suspected of OSA.

**Methods:**

In this study, we collected the basic data and polysomnography (PSG) data of patients diagnosed with hypertension who underwent PSG at the Sleep Medicine Center of the First Affiliated Hospital of Guangzhou Medical University from April 2012 to March 2021. The sensitivity, specificity, positive predictive value, negative predictive value, area under the curv (AUC) and diagnostic odds ratio (DOR) of the six screening tools were then calculated, and their correlation with the sleep apnea hypopnea index (AHI) analyzed.

**Results:**

There were 303 males (303/398, 76.1%) out of 398 hypertension patients suspected of OSA. The area under the curve of the Berlin questionnaire's receiver operating characteristic (ROC) curve reached 0.753 (95%CI: 0.707–0.794). When the AHI was 5, 15 and 30 times/h as the cut‐off points, the sensitivity and negative predictive value of Berlin were the highest at 0.947 and 0.630, 0.970 and 0.851, and 0.988 and 0.957 respectively, while the specificity and positive predictive value of the Epworth Sleepiness Scale (ESS) were the highest at 0.696 and 0.729, 0.750 and 0.887, and 0.674 and 0.575 respectively. The DOR value of the Berlin questionnaire could reach 18.333 when the AHI cut‐off point was 30 times/h. Berlin had the largest rank correlation coefficient with AHI at 0.466.

**Conclusion:**

The Berlin questionnaire can be considered a priority for the screening and stratifying of hypertensive patients suspected of OSA.

**Abbreviations**
AHI: apnea hypopnea index
AUC: area under the curve
BMI: body mass index
CI: confident interval
DOR: diagnostic odds ratio
DP: diastolic pressure
ESS: Epworth Sleepiness Scale
HR: heart rate
Mean‐NOX: mean nocturnal oxygen saturation
Min‐NOX: minimum nocturnal oxygen saturation
NC: neck circumference
NPV: negative predictive value
OSA: obstructive sleep apnea
PPV: positive predictive value
PSG: polysomnography
ROC: receiver operating curve
SDB: sleep disordered breathing
SP: systolic pressure
WC: wrist circumference

## 1 INTRODUCTION

Obstructive sleep apnea (OSA) is a kind of disease in which partial or complete upper airway obstruction occurs repeatedly during sleep, causing a series of clinical manifestations.^1^, ^2^ OSA is associated with many diseases such as hypertension, coronary heart disease, type 2 diabetes, cerebral infarction, and non‐alcoholic fatty liver disease. In particular, moderate to severe OSA can be detected in approximately one‐third or more of patients with primary hypertension.^3^, ^4^ Studies have reported that in 30% of patients with hypertension and up to 80% of patients with drug‐resistant hypertension, AHI ≥15 events will occur every hour, and recurrent OSA will further affect the development of hypertension.^4^ The coexistence and two‐way relationship between OSA and hypertension will increase the risk of cardiovascular disease.^4^ The disease seriously affects the quality of life and health of patients, especially untreated OSA drivers, whose risk of motor vehicle accidents increases significantly. There is no doubt that OSA has become an important public health problem.^5^, ^6^ Therefore, the earlier recognition and treatment of patients with hypertension suspected of OSA is vitally important.

At present, the gold standard for diagnosing OSA is polysomnography (PSG) at night, but this is expensive, time‐consuming and limited to areas with good medical care, which will cause delays in the diagnosis and treatment of a large number of patients with suspected OSA.^7^ In this case, several low‐cost, easy‐to‐operate and acceptable screening tools have been developed, bringing great convenience to the screening of OSA. At present, these mainly include the NoSAS score, No‐Apnea score, Epworth Sleepiness Scale (ESS), STOP questionnaire, STOP‐Bang questionnaire and Berlin questionnaire. The NoSAS score is a new screening tool which was developed in a Swiss cohort of 2121 subjects (HypnoLaus) and subsequently validated in a Brazilian cohort of 1042 subjects (EPISONO).^8^ The No‐Apnea score is a newly developed and validated screening tool that only includes two objective parameters: neck circumference and age.^7^ ESS, which was originally designed to assess the risk of daytime sleepiness, can assess the subjective possibility of falling asleep in various environments, and was subsequently recommended as a tool for identifying OSA.^9^ The STOP questionnaire is a self‐report designed by anesthesiologists and sleep experts based on the Berlin questionnaire and literature reviews.^10^ The STOP‐Bang questionnaire has become a widely used tool in OSA testing. It was initially used to screen surgical patients, but later proved to be a high‐quality method with good consistency in identifying the severity of OSA in patients.^9^, ^11^ The Berlin questionnaire was developed by a group of respiratory and primary care doctors in Germany in 1996 through consensus, and has become a qualitative diagnostic tool for OSA that is widely used in the world.^12^ In the design of some of these scales, hypertension is considered a risk factor for OSA. It has also been reported that OSA can affect the fluctuation of nocturnal blood pressure.^4^ At present, these screening tools are used to screen OSA, but the specificity, sensitivity, predictive value, receiver operating characteristic (ROC) curve analysis and diagnostic odds ratio (DOR) of each screening tool are different. Thus, we compared the application value of these six screening tools in patients with hypertension suspected of OSA in order to find a more suitable scale. We hypothesize that the screening ability of a scale with a hypertension option will be relatively high in patients with hypertension.

## 2 METHODS

### 2.1 Study subjects

This study collected data on patients diagnosed with hypertension who underwent PSG examination at the Sleep Medicine Center of the First Affiliated Hospital of Guangzhou Medical University from April 2012 to March 2021. If a patient had a clear history of hypertension or was taking antihypertensive drugs, or they had systolic blood pressure ≥ 140 mmHg or diastolic blood pressure ≥ 90 mmHg before and after monitoring, they were considered hypertensive. This study was approved by the medical ethics committee of the hospital (Ethics Number: 201705), and eligible patients were selected according to the inclusion and exclusion criteria. Inclusion criteria: (a) Patients who first came to the sleep respiratory center for PSG monitoring due to complaints of snoring, lethargy, high blood pressure or apnea; (b) Patients diagnosed with hypertension; (c) Patients aged between 18 and 80 years old (including 18 and 80 years old); (d) Patients who had autonomous behavioral ability and cognitive ability, had completed six scales in the sleep laboratory and agreed to sign the informed consent; (e) Patients with total sleep time > 4 h. Exclusion criteria: (a) Patients with coronary heart disease, diabetes, kidney disease, chronic lung disease, or cerebrovascular disease; (b) Patients with a history of brain tumors or epilepsy; (c) Patients with various mental and psychological diseases who were taking sedative and sleeping pills; (d) Patients with severe organ failure; (e) OSA patients who had received treatment; (f) Patients with incomplete answers on the scale; (g) Patients with sleep apnea hypopnea syndrome with predominantly central or mixed events.

### 2.2 Basic data collection

In our study, we collected general information such as the patients' name, age, gender, occupation, education, smoking, drinking, height, weight, neck circumference, waist circumference, blood pressure, and so on. The patient and family members filled in the ESS, STOP, STOP‐Bang and Berlin scales together, and the sleep technician verified the items on the scales to ensure their reliability, then took back the completed questionnaires. The researchers then refined the NoSAS and No‐Apnea scores based on the general data.

### 2.3 Questionnaire

1. NOSAS^8^: The score is 0–17 points, including five questions: ① Neck circumference (NC) > 40 cm is 4 points; ② 25 < Body mass index (BMI) < 30 kg/m^2^ is 3 points, BMI ≥30 kg/m2 is 5 points; ③ Snoring is 2 points; ④ Age ≥ 55 years old is 4 points; ⑤ Male is 2 points. If the NoSAS score ≥ 8 points, it indicates that the patient is at high risk of OSA.
2. No‐Apnea^7^: The score is 0–9 points, including two variables: ① Neck circumference (NC) < 37.0 is 0 points, 37.0–39.9 is 1 point, 40.0–42.9 is 3 points, NC ≥43.0 is 6 points; ② Age < 35 is 0 points, 35–44 is 1 point, 45–54 is 2 points, ≥ 55 years old is 3 points. If the No‐Apnea score is ≥3 points, it indicates that the patient is at high risk of OSA.
3. ESS^9^: Including 8 questions, the subjects are asked to evaluate the degree of dozing in a specific scenario during the day; 0 is no dozing and 1, 2, and 3 are light, moderate and severe dozing. The total score is 24 points. If the ESS score is ≥9 points, there is daytime sleepiness.
4. STOP^10^: Including 4 problems, namely snoring, fatigue, observed apnea and high blood pressure. Answer with “yes” or “no”; “yes” is 1 point, “no” is 0 points. If the score of the 4 questions is >2, it indicates that the patient is at high risk of OSA.
5. STOP‐Bang^9^, ^11^: On the basis of the STOP scale, add “bang”, namely B [body mass index (BMI) > 35 kg/m^2^], A (age > 50 years old), N (neck circumference > 40 cm), G (male). Answer with “yes” or “no”; “yes” is 1 point, “no” is 0 points. If the STOP‐Bang score is ≥3 points, it indicates that the patient is at high risk of OSA.
6. Berlin^12^: There are 11 problems in 3 groups: ① severity of snoring; ② daytime sleepiness; ③ high blood pressure or obesity. Each group is evaluated as negative or positive after calculating the score. If two or more of the 3 groups are positive, the patient is considered to have a high risk of OSA (high‐risk group). If only one or none of the 3 groups is positive, the patient is considered to have a low risk of apnea (low‐risk group).

### 2.4 Polysomnography (PSG)

PSG monitoring was mainly used to diagnose sleep disordered breathing. Recording indicators include electroencephalogram, electrooculogram, mandibular electromyography, oral and nasal airflow and respiratory movement, electrocardiogram, blood oxygen saturation, snoring, limb movement, body position and other parameters. We used an Alice 5 polysomnograph made by the Philips Wellcome Company to record continuously and synchronously for at least 7 h. After automatic analysis, the original parameters were manually reviewed and corrected, and finally interpreted and analyzed by trained sleep physicians. Sleep apnea hypopnea index (AHI) refers to the number of apnea and hypopnea events per hour of sleep. Patients with AHI ≥5 times/h and obstructive apneas as the main respiratory event were judged to have OSA in the following disease grades: normal group (AHI < 5 times/h), mild OSA group (5 ≤ AHI < 15 times/h), moderate OSA group (15 ≤ AHI < 30 times/h), severe OSA group (AHI ≥30 times/h).^4^

### 2.5 Statistical analysis

SPSS 26.0 statistical software was used for analysis. The measurement data of normal distribution were expressed as mean ± SD, the measurement data of skewed distribution was expressed as median (25th, 75th quantile) (M [P25, P75]) and the count data was expressed as frequency. For the measurement data, a one‐way analysis of variance test was used for normal distribution, a multi‐group independent sample rank test (Kruskal‐Wallis one‐way ANOVA (k) (w) multiple comparison) was used for skewed distribution data, and a chi‐square test or Fisher's exact probability method was used to count the data. The diagnostic results of the scales and PSG were calculated in the form of a four‐grid table for the sensitivity, specificity, positive predictive value (PPV), negative predictive value (NPV), positive likelihood ratio (LR+), and negative likelihood ratio (LR‐) of each scale, and reported with respective 95% confidence intervals (CI). The ROC curve was analyzed using MedCalc software to evaluate the diagnostic value of the five scales for OSA, and calculate the *p* value for comparing the area under the ROC curve of single and multiple indicators. The correlation between the six scales and AHI was analyzed using the bivariate correlation method. Pearson correlation analysis was used for the normal distribution data, and Spearman correlation analysis was used for the non‐normal distribution data. *p* < .05 was defined as statistically significant.

## 3 RESULTS

### 3.1 Baseline characteristics

According to our inclusion and exclusion criteria, among the 398 suspected OSA patients with hypertension collected from the Sleep Medicine Center, 303 were male (303/398, 76.1%), 152 were smokers (152/398, 38.2%) and 105 cases were drinkers (105/398, 26.4%). Among them, the gender difference was statistically significant (*p* < .001) with the disease predominantly affecting males, but there was no statistically significant difference for smoking and drinking. The average age of these patients was (51.8 ± 12.5) years old, average systolic blood pressure was (149.2 ± 17.6) mmHg, average diastolic blood pressure was (95.9 ± 11.6) mmHg, average heart rate was (79.4 ± 12.2) beats/min, average body mass index was (27.6 ± 3.8) kg/m^2^, average neck circumference was (39.4 ± 3.8) cm and average waist circumference was (98.6 ± 10.7) cm. There were no statistically significant differences in age, systolic blood pressure or diastolic blood pressure, but differences in heart rate, body mass index, neck circumference and waist circumference were statistically significant (*p* < .001). The medians of NoSAS, No‐Apnea, ESS, STOP, STOP‐Bang, Berlin, AHI, lowest nocturnal oxygen saturation, and mean nocturnal oxygen saturation were respectively 11 (7, 13) points, 4 (3, 6) points, 8 (3, 12.8) points, 3 (2, 3) points, 5 (4, 6) points, 2 (2, 3) points, 19.7 times/h, (7.6, 50.7), 80% (68, 86) and 94% (71, 96), with statistically significant differences (*p* < .001). In the post test, except for drinking, age, systolic blood pressure and diastolic blood pressure, there were significant differences among the severe OSA group and normal group, and the mild OSA group and moderate OSA group respectively (*p* < .005) (Table 1).

**TABLE 1:** Baseline characteristics of study subjects

Project	ALL	Normal group	Mild OSA	Moderate OSA	Severe OSA	p1	p2	p3	p4	p5	p6	p7
Number of cases (%)^a^	398	76 (19.1)	85 (21.4)	75 (18.8%)	163 (41)	—	—	—	—	—	—	—
Male (%)^a^	303 (76.1)	52 (68.4)	59 (69.4)	51 (68)	141 (86.5)	<.001	.892	.956	.001	.848	.001	.001
Smoking (%)^a^	152 (38.2)	29 (38.1)	28 (32.9)	27 (36.0)	68 (41.7)	.57	<.001	<.001	<.001	<.001	<.001	<.001
Drinking (%)^a^	105 (26.4)	15 (19.7)	27 (31.8)	18 (24.0)	46 (28.2)	.446	<.001	<.001	<.001	<.001	<.001	<.001
Age (years old)^b^	51.8±12.5	54.4±13.3	52.0±11.5	51.2±14.1	50.8±11.9	.273	.235	.114.	.039	.660	.456	.829
SP (mmHg)^b^	149.2±17.6	151±18.2	146.6±17.6	149.3±17.2	149.5±17.6	.428	.107	.525	.525	.340	.215	.915
DP (mmHg)^b^	95.9±11.6	95.3±10.0	94.0±10.0	95.4±11.8	97.5±12.8	.115	.484	.936	.157	.435	.022	.188
HR (events/min)^b^	79.4±12.2	80.9±12.7	75.9±11.9	76.5±11.8	81.8±11.8	<.001	.008	.024	.573	.754	<.001	.001
BMI (kg/m^2^)^b^	27.6±3.8	26.5±3.9	26.4±3.0	27.4±3.4	28.7±4.1	<.001	.829	.112	<.001	.065	<.001	.010
NC (cm)^b^	39.4±3.8	38.2±4.2	37.9±3.2	39.0±3.0	40.8±3.8	<.001	.610	.183	<.001	.061	<.001	<.001
WC (cm)^b^	98.6±10.7	94.9±10.7	95.3±8.8	96.6±8.6	103.0±10.9	<.001	.810	.316	<.001	.429	<.001	<.001
NoSAS (points)^c^	11 (7,13)	8 (6,11)	8 (7,11)	11 (7,12)	11 (9,13)	<.001	.896	.123	<.001	.086	<.001	.002
No‐apnea(points)^c^	4 (3,6)	3 (3,5.8)	3 (3,5)	4 (3,6)	5 (3,6)	<.001	.473	.593	<.001	.206	<.001	<.001
ESS(points)^c^	8 (3,12.8)	5 (3,8.8)	6 (2,10)	6 (3,10)	11(5,15)	<.001	.591	.364	<.001	.691	<.001	<.001
STOP(points)^c^	3 (2,3)	2 (2,3)	2 (2,3)	3 (2,3)	3 (3,4)	<.001	.018	<.001	<.001	.144	<.001	.001
STOP‐bang (points)^c^	5 (4,6)	4 (3,5)	4 (4,5)	5 (4,5)	5 (5,6)	<.001	.233	.004	<.001	.072	<.001	<.001
Berlin(points)^c^	2 (2,3)	2 (1,2)	2 (2,3)	2 (2,3)	2 (2,3)	<.001	<.001	<.001	<.001	.096	<.001	.005
AHI (events/h)^c^	19.7 (7.6,50.7)	2.4 (1,3.8)	9.5 (7.5,11.4)	19.6 (17.3,22.6)	56.8 (41.9,68.0)	<.001	<.001	<.001	<.001	<.001	<.001	<.001
Min‐NOX (%)^c^	80 (68,86)	89 (85.3,91)	84 (79.5,87)	80 (74,83)	68 (56,77)	<.001	<.001	<.001	<.001	.001	<.001	<.001
Mean‐NOX (%)^c^	94 (71,96)	96 (95,97)	96 (95,97)	95 (94,96)	68 (56,77)	<.001	.909	.243	<.001	.189	<.001	<.001

*Note*: p, p value; p1 is the comparison of the four groups together; p2 is the comparison between the normal group and the mild OSA group; p3 is the comparison between the normal group and the moderate OSA group; p4 is the comparison between the normal group and the severe OSA group; p5 is the comparison between mild OSA group and moderate OSA group; p6 is the comparison between the mild OSA group and the severe OSA group; p7 is the comparison between the moderate OSA group and the severe OSA group.

Abbreviations: AHI, apnea hypopnea index; BMI, body mass index; DP, diastolic pressure; ESS, Epworth Sleepiness Scale; HR, heart rate; Mean‐NOX, mean nocturnal oxygen saturation; Min‐NOX, minimum nocturnal oxygen saturation; NC, neck circumference; SP, systolic pressure; WC, wrist circumference.

^a^ Frequency and Chi‐square test.

^b^ Mean and variance and one‐way ANOVA.

^c^ Median and rank sum test.

### 3.2 Predictive value of six scales

**FIGURE 1:**
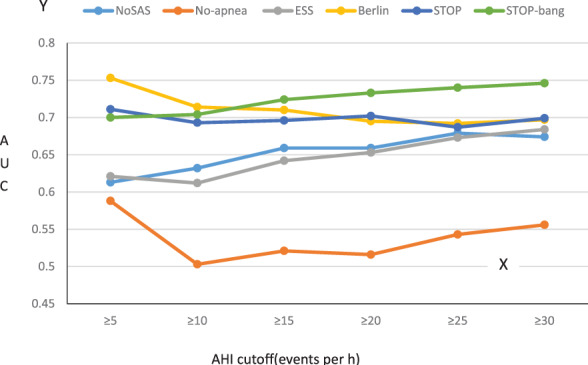
Predictive value of six scales at different AHI cut‐off points. The area under the ROC curve of the six scales was compared with the cut‐off point of AHI of 5, 10, 15, 20, 25, and 30 times/h. AHI, apnea‐hypopnea index; AUC, area under the curve; ESS, Epworth Sleepiness Scale

**FIGURE 2:**
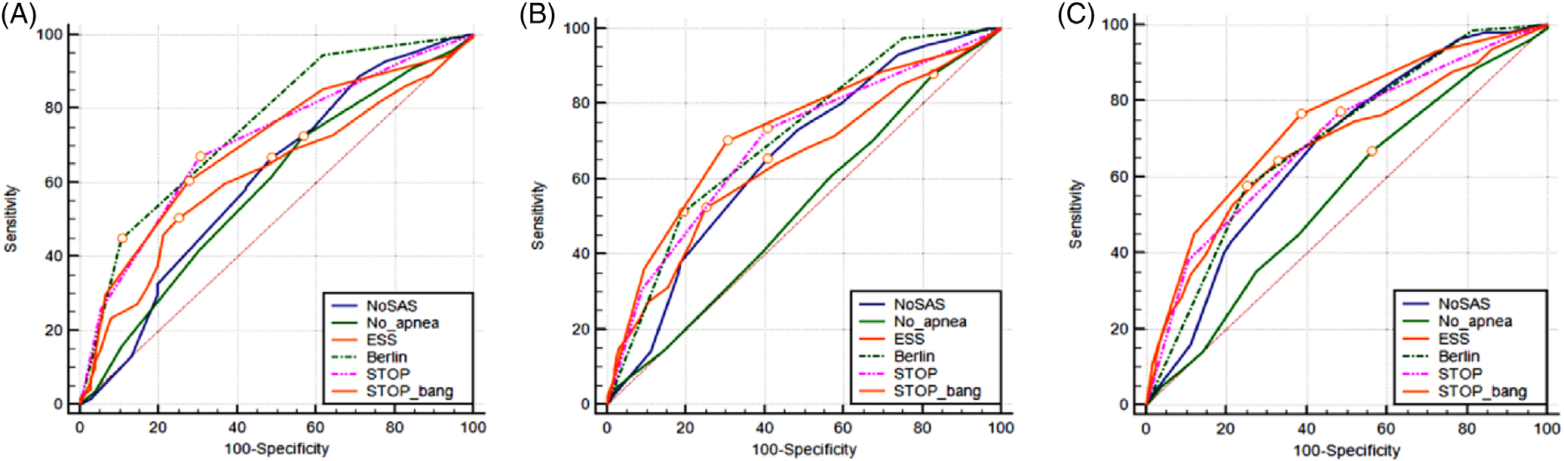
ROC curve of the six screening tools at AHI cutoff of ≥5, 15 and 30 events/h. (A) ROC curve of the six screening tools at AHI cutoff of ≥5 events/h; (B) ROC curve of the six screening tools at AHI cutoff of ≥15 events/h; ROC curve of the six screening tools at AHI cutoff of ≥30 events/h. AHI, apnea hypopnea index; ESS, Epworth Sleepiness Scale; ROC, receiver operating curve

The areas under the curve of the ROC of the six scales were compared with AHI of 5, 10, 15, 20, 25, and 30 times/h as the cut‐off points^5^ (Figure 1). Obviously, the area under the curve of No‐Apnea's ROC was below 0.600, and the diagnostic value was low. When AHI ≥5 was used as the diagnostic criteria for OSA, the area under the curve of Berlin's ROC was 0.753 (95%CI: 0.707–0.794), giving it the highest predictive value, but then its predictive value gradually decreased. When AHI was 5, 10, 15, 20, 25, and 30 times/h as the cut‐off points, the areas under the curve of STOP‐Bang's ROC were 0.700 (95%CI: 0.652–0.744), 0.704 (95%CI: 0.657–0.749), 0.724 (95%CI: 0.678–0.768), 0.733 (95%CI: 0.687–0.776), 0.740 (95%CI: 0.694–0.783) and 0.746 (95%CI: 0.700–0.788) respectively, with statistically significant differences (*p* < .001). When AHI was 20, 25, and 30 times/h as the cut‐off point, the predictive value of STOP‐Bang was significantly better than those of the other five scales with statistically significant difference (*p* < .005) (Figure 2).

### 3.3 Predictive indicators of six scales

When AHI was 5, 15, and 30 times/h as the cut‐off points, Berlin had the highest sensitivity and negative predictive value of 0.947 and 0.630, 0.970 and 0.851, and 0.988 and 0.957 respectively, while its specificity and positive predictive value were 0.382 and 0.866, 0.248 and 0.655, and 0.186 and 0.455 respectively. When AHI was 5, 15, and 30 times/h as the cut‐off points, the specificity and positive predictive value of ESS were the highest at 0.696 and 0.729, 0.750 and 0.887, and 0.674 and 0.575 respectively, while its sensitivity and negative predictive value were 0.463 and 0.248, 0.557 and 0.516, and 0.642 and 0.733 respectively. When AHI was 5, 15, and 30 times/h as the cut‐off points, the sensitivity and negative predictive value of STOP‐Bang were 0.944 and 0.217, 0.954 and 0.522, and 0.981 and 0.870 respectively, and its specificity and positive predictive value were 0.066 and 0.811, 0.075 and 0.603, and 0.085 and 0.424 respectively (Table 2).

**TABLE 2:** With cut‐off point of AHI of 5, 15, and 30 as the diagnose of OSA disease (95%CI) (95%CI)

Questionnaire	AUC	Sensitivity	Specificity	PPV	NPV	DOR
With cut‐off point of AHI of 5						
NoSAS	0.613 (0.563‐0.661)	0.748 (0.701‐0.796)	0.408 (0.298‐0.518)	0.843 (0.800‐0.885)	0.277 (0.194‐0.360)	2.050 (1.216‐3.455)^a^
No‐apnea	0.558 (0.538‐0.637)	0.842 (0.802‐0.881)	0.105 (0.036‐0.174)	0.799 (0.757‐0.842)	0.136 (0.048‐0.223)	0.625 (0.283‐1.379)
ESS	0.621 (0.571‐0.669)	0.463 (0.408‐0.517)	0.750 (0.653‐0.847)	0.887 (0.839‐0.935)	0.248 (0.192‐0.30)	2.584 (1.471‐4.539)^a^
Berlin	0.753 (0.6707‐0.794)	0.947 (0.923‐0.972)	0.382 (0.272‐0.490)	0.866 (0.831‐0.902)	0.630 (0.491‐0.770)	11.070 (5.648‐21.696)^a^
STOP	0.711 (0.664‐0.755)	0.947 (0.923‐0.972)	0.145 (0.066‐0.224)	0.824 (0.786‐0.863)	0.393 (0.212‐0.574)	3.036 (1.358‐6.786)^a^
STOP‐bang	0.700 (0.652‐0.744)	0.944 (0.920‐0.969)	0.066 (0.010‐0.122)	0.811 (0.771‐0.850)	0.217 (0.049‐0.386)	1.189 (0.427‐3.311)^a^
With cut‐off point of AHI of 15						
NoSAS	0.659 (0.610‐0.705)	0.802 (0.751‐0.852)	0.403 (0.327‐0.480)	0.664 (0.609‐0.720)	0.580 (0.489‐0.672)	2.737 (1.748‐4.286)^a^
No‐apnea	0.521 (0.471‐0.571)	0.852 (0.807‐0.890)	0.596 (0.554‐0.649)	0.596 (0.544‐0.648)	0.407 (0.281‐0.532)	1.011 (0.576‐1.775)
ESS	0.642 (0.593‐0.689)	0.557 (0.494‐0.620)	0.696 (0.625‐0.767)	0.729 (0.665‐0.794)	0.516 (0.450‐0.583)	2.873 (1.884‐4.384)^a^
Berlin	0.710 (0.662‐0.754)	0.970 (0.949‐0.922)	0.248 (0.181‐0.315)	0.655 (0.606‐0.705)	0.851 (0.749‐0.953)	10.862 (4.724‐24.975)^a^
STOP	0.696 (0.648‐0.741)	0.954 (0.927‐0.980)	0.156 (0.058‐0.153)	0.611 (0.561‐0.660)	0.607 (0.426‐0.788)	2.426 (1.104,5.327)^a^
STOP‐bang	0.724 (0.678‐0.768)	0.954 (0.927‐0.980)	0.075 (0.033‐0.115)	0.603 (0.553‐0.652)	0.522 (0.318‐0.726)	1.655 (0.712‐3.848)
With cut‐off point of AHI of 30						
NoSAS	0.674 (0.625‐0.720)	0.858 (0.804‐0.912)	0.377 (0.315‐0.439)	0.486 (0.428‐0.544)	0.795 (0.720‐0.869)	3.659 (2.189‐6.116)^a^
No‐apnea	0.556 (0.506‐0.605)	0.889 (0.840‐0.937)	0.173 (0.125‐0.222)	0.425 (0.372‐0.477)	0.695 (0.577‐0.812)	1.682 (0.928‐3.048)
ESS	0.684 (0.636‐0.729)	0.642 (0.568‐0.716)	0.674 (0.614‐0.734)	0.575 (0.502‐0.647)	0.733 (0.674‐0.792)	3.703 (2.430‐5.641)^a^
Berlin	0.697 (0.649‐0.742)	0.988 (0.971‐1.005)	0.186 (0.137‐0.236)	0.455 (0.403‐0.507)	0.957 (0.898‐1.015)	18.333 (4.376‐76.801)^a^
STOP	0.699 (0.651‐0.744)	0.969 (0.943‐0.996)	0.097 (0.060‐0.135)	0.424 (0.374‐0.475)	0.821 (0.680‐0.963)	3.391 (1.261‐9.114)^a^
STOP‐bang	0.746 (0.700‐0.788)	0.981 (0.961‐1.002)	0.085 (0.049‐0.120)	0.424 (0.374‐1.007)	0.870 (0.732‐1.007)	4.907 (1.433‐16.800)^a^

Abbreviations: AUC, the area under the ROC curve; CI, confident interval; DOR, diagnostic odds ratio; ESS, Epworth Sleepiness Scale; NPV, negative predictive value; PPV, positive predictive value.

^a^ *p* < .05.

### 3.4 Judgment of correctness of six scales in diagnosis of OSA

Relevant studies have shown that it is a good method to compare the accuracy of various OSA prediction models and questionnaires with the diagnostic odds ratio.^13^ When AHI was 5 times/h as the cut‐off point, the DOR values of NoSAS, No‐Apnea, ESS, STOP, STOP‐Bang and Berlin were 2.050, 0.625, 2.584, 3.036, 1.189, and 11.070 respectively. When AHI was 15 times/h as the cut‐off point, the DOR values of NoSAS, No‐Apnea, ESS, STOP, STOP‐Bang and Berlin were 2.737, 1.011, 2.873, 2.426, 1.655, and 10.862 respectively. When AHI was 30 times/h as the cut‐off point, the DOR values of NoSAS, No‐Apnea, ESS, STOP, STOP‐Bang, and Berlin were 3.659, 1.682, 3.703, 3.391, 4.907, and 18.333 respectively. When AHI was 5 times/h as the cut‐off point, the STOP‐Bang difference was not statistically significant. When AHI was 5, 15, and 30 times/h as the cut‐off point, the No‐Apnea difference was not statistically significant, and all other differences were statistically significant (Table 2).

### 3.5 Correlation analysis between six scales and AHI

According to the correlation analysis of the six scales and AHI, it is clear that because the scores of the six scales were non‐normally distributed, rank correlation analysis was used. The rank correlation coefficients of NoSAS, No‐Apnea, ESS, STOP, STOP‐Bang, and Berlin were 0.296, 0.043, 0.329, 0.424, 0.453, and 0.466 respectively. Among them, the *p* value of no‐apnea was .397 with no statistically significant difference, and the *p* values of the other five scales were less than .001 with statistically significant differences (Table 3).

**TABLE 3:** Correlation analysis between the scores of each scale and AHI

Group	NoSAS	No‐apnea	ESS	STOP	STOP‐bang	Berlin
AHI	0.296^a^	0.043	0.329^a^	0.424^a^	0.453^a^	0.466^a^
*p*	<.001	.397	<.001	<.001	<.001	<.001

Abbreviations: AHI, apnea hypopnea index; ESS, Epworth Sleepiness Scale.

^a^ *p* < .01.

## 4 DISCUSSION

OSA is the most common sleep disordered breathing disease, as well as a disease with a high incidence and low diagnosis rate. According to the relevant research reports, the prevalence of OSA is rising rapidly, which may be due to the combined effect of various factors such as the obesity epidemic, increased sensitivity of sleep study recording sensors and reduced stringency after updated scoring standards,^12^ which is also related to the various high‐risk factors of the disease. In our study, 322 out of 398 hypertensive patients suspected of OSA were diagnosed. The proportion of males was much higher than that of females, which is consistent with the results of previous epidemiological studies.^3^, ^14^ It has been reported in the literature that the patient's neck circumference, waist circumference and BMI value affect the severity of OSA,^15^ which is also consistent with our research results. However, our research shows that the differences between OSA and smoking, drinking and age were not statistically significant. In addition, related studies have shown that neck circumference, waist circumference and BMI are also risk factors for hypertension.^16^ It can be seen that high‐risk factors such as gender, neck circumference, waist circumference and BMI value will affect the identification and severity distribution of OSA patients with hypertension on the screening form.

The purpose of this study was to compare the application value of six screening tools in the screening of OSA in patients with hypertension. From the distribution of the ROC curve, although the area under the curve of No‐Apnea's ROC increased with the increase in AHI, the area under the curve of No‐Apnea's ROC was lower and almost less than 0.6, which may be because No‐Apnea only includes the two objective parameters of neck circumference and age, while there are many risk factors for both OSA and hypertension. In addition, the sensitivity and negative predictive value of No‐Apnea are lower than those of STOP‐Bang and Berlin. Therefore, No‐Apnea has low efficacy in screening for OSA in hypertensive patients, and this new screening tool still needs further verification.

The ideal screening tool should have high sensitivity and specificity at the same cut‐off value, but this is a very rare case. However, the sensitivity and specificity of the screening model are usually inversely correlated, and high sensitivity is often gained at the expense of specificity.^7^, ^11^ For diseases such as OSA combined with hypertension, it may be more important that the screening test has high sensitivity so as not to miss OSA patients rather than having high specificity.^17^ The Berlin questionnaire classifies patients as high‐risk or low‐risk based on self‐reports of snoring, daytime sleepiness, hypertension and obesity, with different sensitivity and specificity in different studies.^18^ In this study, when AHI was cut off at 5 times/h, compared with the other five screening tools, the area under the curve of Berlin's ROC was the largest at 0.753, and even if it showed a downward trend, the area under the curve of Berlin's ROC was still 0.697 when AHI was cut off at 30 times/h. Most importantly, among the six screening tools, the sensitivity and negative predictive value of Berlin were the highest, and increased with the increase in OSA degree. They were highest when AHI was 30 times/h as the cut‐off point, reaching 0.988 and 0.957. This is similar to the research results of Tan A et al.^12^ Therefore, Berlin is of great value for the screening of OSA in hypertensive patients. However, it has more contents, which is a factor that we should consider as it may affect our clinical application.

People with a higher prevalence of OSA may have more symptoms or associated comorbidities, resulting in higher sensitivity.^12^ Due to the practicality and high sensitivity of STOP‐Bang, it has been widely used in the world. In a surgical environment, the sensitivity of STOP‐Bang score ≥ 3 in patients with mild, moderate and severe OSA was 84%, 93%, and 100%, respectively.^6^ In our study, the area under the curve of STOP‐Bang's ROC was always above 0.7 and showed an upward trend. The sensitivity and negative predictive value increased with the increase in OSA degree (AHI from 5 to 30 times/h), reaching highest points of 0.981 and 0.870, which means it has a high predictive value for the screening of OSA in hypertensive patients. Chiu H Y et al.^19^ conducted a two‐factor meta‐analysis of 108 studies and found that compared with BQ, STOP and ESS, STOP‐Bang is a more accurate tool for detecting mild, moderate and severe OSA. Like STOP‐Bang, STOP also has higher sensitivity and negative predictive value, but not as high as those of the STOP‐Bang questionnaire, which also proves that the improved STOP‐Bang questionnaire based on the STOP questionnaire is a more effective tool for screening OSA risk.

Our results show that ESS had the highest specificity and positive predictive value among the six screening tools, but its sensitivity and negative predictive value were relatively low. Duarte R et al.^20^ found that ESS had the highest specificity but low sensitivity, which is consistent with our research results. Many reports in the literature also state that ESS is inferior to other screening tools in identifying high‐risk patients with OSA.^9^, ^19^, ^21^, ^22^ Perhaps this is because the ESS score was originally designed to assess the risk of daytime sleepiness. Therefore, ESS is not effective as a screening tool for patients with hypertension suspected of OSA. Some studies have found that the areas under the curve of STOP‐Bang and NoSAS's ROCs were always very high, making them powerful tools for the screening and stratification of OSA patients, but the diagnostic ability of STOP‐Bang is higher than that of NoSAS.^14^ However, on the contrary, Rong et al.^23^ found that NoSAS has good predictive value for screening patients with sleep disorders, and its discrimination ability is higher than that of STOP‐Bang. More studies have reported that NoSAS shows better discrimination ability than the ESS, Berlin and STOP‐Bang screening tools, not only in moderate to severe sleep disordered breathing, but also in mild cases.^24^ In our study, NoSAS performed poorly, and was worse than STOP, STOP‐Bang and Berlin in identifying hypertensive patients suspected of OSA. Therefore, as a new screening tool, NoSAS requires further verification.

In our study, in order to further compare the accuracy of the application value of the six screening tools, we conducted an analysis of the diagnostic odds ratio and its correlation with AHI. The DOR represents the best single‐point estimate of the ROC curve, which importantly has nothing to do with epidemics but provides a decision‐making tool for doctors to distinguish between healthy and unhealthy patients.^13^ The greater the DOR, the better the accuracy of the diagnosis.^13^ We found that the DOR values of the six screening tools all increased with the degree of OSA, especially the Berlin questionnaire which had DOR values all above 10, reaching 18.333 when the AHI was 30 times/h as the cut‐off point. After the correlation analysis between the six screening tools and AHI, the correlation coefficient between Berlin and AHI was also the largest, which shows that Berlin was significantly better than the other five screening tools at identifying hypertensive patients suspected of OSA, and its ability becomes stronger as the degree of OSA combined with hypertension intensifies. At present, several low‐cost, easy‐to‐operate and acceptable screening tools have been developed, but they have different levels of ability to screen for OSA in different diseases, and it is impossible to answer every scale in clinical practice. Our research is intended to identify a more suitable scale for screening hypertensive patients suspected of OSA.

### 4.1 Limitations

First, this was a retrospective single‐center study. Generally speaking, the use of retrospective analysis to verify the predictive value of different screening tools is not as ideal as prospective research. However, the indicators that needed to be used in the research were included in the questionnaire survey of every patient undergoing PSG examination at our center, which basically solves the limitations of retrospective analysis. Second, the sample size was relatively small, predominantly male and aged around 50, which may have restricted our research results, and further study is required to evaluate effectiveness in younger people. Third, in general, patients who come to the sleep laboratory due to main complaints such as snoring, sleepiness, hypertension or apnea are usually suspected of OSA, and patients with hypertension may obtain higher results in the selected scale compared with non‐hypertensive individuals, which virtually increases the probability of diagnosis. Fourth, this study may be affected by regional distribution (Asian population), so it needs to be further verified in other environments.

## 5 CONCLUSIONS

In conclusion, for screening hypertensive patients suspected of OSA, although there are many test contents in Berlin, relatively speaking, it is still better than the other five screening tools, and can be considered a priority for the screening and stratification of hypertensive patients suspected of OSA. The screening of STOP‐Bang is better than that of STOP, NoSAS and ESS, and it can also be used to screen for hypertensive patients suspected of OSA. NoSAS is simple to use but requires further certification as a new screening tool. Finally, ESS is not effective and no‐apnea may not be suitable in screening for hypertensive patients suspected of OSA.

## CONFLICT OF INTEREST

The authors declare no conflicts of interest.

## Data Availability

All data generated or analysed during this study are included in this published article.
